# Endotoxin Activity Assay-guided Polymyxin B Hemoperfusion: From Failed Trials to Biological Enrichment

**DOI:** 10.14789/ejmj.JMJ26-0042-R

**Published:** 2026-06-30

**Authors:** RICARD FERRER, KOKI NAMEKAWA, SHUNICHIRO URABE, HIROSHI MUKAIDA, NAO UMEI, TOSHIAKI IBA

**Affiliations:** 1 Intensive Care Department, Hospital Universitari Vall d’Hebron Universitat Autònoma de Barcelona, Barcelona, Spain; 1 Intensive Care Department, Hospital Universitari Vall d’Hebron Universitat Autònoma de Barcelona, Barcelona, Spain; 2 Faculty of Medical Science, Juntendo University, Chiba, Japan; 2 Faculty of Medical Science, Juntendo University, Chiba, Japan; 3 Department of Emergency and Disaster Medicine, Juntendo University Graduate School of Medicine, Tokyo, Japan; 3 Department of Emergency and Disaster Medicine, Juntendo University Graduate School of Medicine, Tokyo, Japan

**Keywords:** sepsis, shock, biomarker, precision medicine, randomized controlled trial

## Abstract

Septic shock remains a leading cause of mortality despite advances in critical care. Polymyxin B hemoperfusion (PMX-HP) has long been proposed to improve outcomes by removing circulating endotoxin, yet randomized trials have yielded inconsistent results. Early enthusiasm from EUPHAS was followed by neutral findings in ABDO-MIX and EUPHRATES, highlighting the limitations of non-enriched trial designs. The latest evidence from the TIGRIS trial, a randomized controlled trial, provides important new insight. In patients with endotoxic septic shock, defined by an endotoxin activity assay (EAA) of 0.60-0.89 and high organ dysfunction, polymyxin B hemoadsorption was associated with a high posterior probability of mortality reduction, particularly at 90 days. This article critically appraises the rationale and limitations of EAA-guided PMX-HP in light of these updated data. While the findings support a biologically enriched strategy, important uncertainties remain regarding assay performance, feasibility, and generalizability. The integration of EAA with complementary biomarkers and clinical phenotypes is likely necessary to advance precision medicine in sepsis.

## Introduction: Early history of polymyxin B hemoperfusion

Septic shock remains a major cause of mortality worldwide, with limited therapeutic options beyond antimicrobial therapy and supportive care. Among adjunctive approaches, polymyxin B hemoperfusion (PMX-HP) was developed to selectively remove circulating endotoxin, a key driver of innate immune activation and endothelial injury in Gram-negative sepsis. Introduced in Japan in the 1990s, PMX-HP rapidly gained clinical interest based on its mechanistic rationale and early observational data suggesting improvements in hemodynamics and organ function.

Subsequent small randomized studies, most notably the EUPHAS trial, reported survival benefit in patients with abdominal septic shock^1)^. However, these promising findings were not consistently reproduced in later multicenter trials. The ABDO- MIX trial failed to demonstrate a mortality benefit, raising concerns regarding treatment timing, patient selection, and protocol adherence^2)^. Similarly, the larger EUPHRATES trial, which incorporated endotoxin activity assay (EAA)-based enrollment (EAA ≥ 0.60), did not show overall survival benefit^3)^.

**Figure 1:**
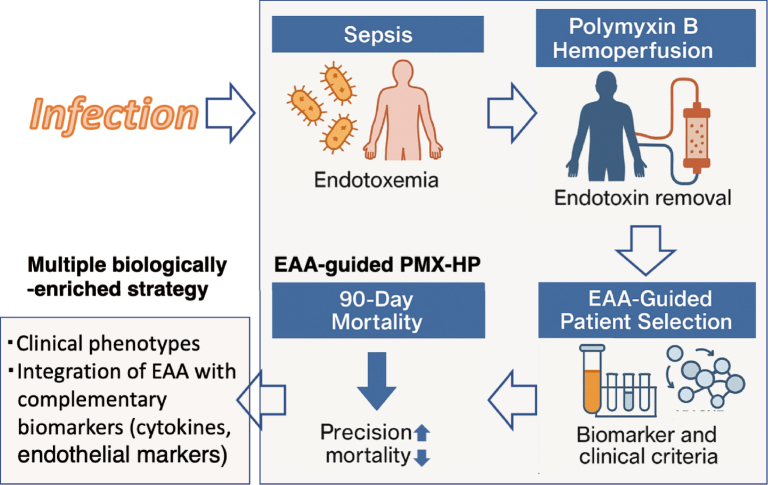
Conceptual framework of endotoxin activity assay (EAA)-guided polymyxin B hemoperfusion (PMX-HP) in septic shock The schematic illustrates the precision medicine concept underpinning EAA-guided PMX-HP. In sepsis, Gram-negative bacteria release endotoxin into the circulation, triggering systemic inflammation and organ dysfunction. The EAA quantifies functional endotoxin activity through neutrophil oxidative burst, allowing biomarker-based patient stratification. Patients with intermediate EAA values (0.60-0.89) and high organ failure scores (e.g., MODS, SOFA) are considered optimal candidates for PMX-HP, which selectively removes circulating endotoxin via a polymyxin B-immobilized cartridge. The TIGRIS trial demonstrated reduced 90-day mortality in this enriched subgroup, validating the biomarker-driven approach. This illustration emphasizes the transition from empiric to biologically targeted therapy in sepsis management through integrated clinical and molecular criteria.

These discordant results highlighted a critical limitation in earlier trial design, namely, the inclusion of biologically heterogeneous populations^4)^. Increasing evidence suggests that endotoxemia varies substantially across septic shock patients and that therapeutic responsiveness depends on both endotoxin burden and host response^5)^. This recognition has shifted the field toward biomarker- guided strategies, setting the stage for subsequent biomarker enrichment-based trials (Figure 1).

## Lessons learned: From EUPHAS to TIGRIS

Successive trials have shaped the clinical trajectory of PMX-HP. As aforementioned, the EUPHAS trial suggested a survival benefit in abdominal septic shock^1)^, whereas ABDO-MIX failed to confirm this effect^2)^. The EUPHRATES trial, which incorporated EAA ≥ 0.60, did not demonstrate an overall mortality benefit^3)^, although post hoc analyses identified a potentially responsive subgroup with intermediate EAA (0.60-0.89) and high disease severity^6)^. These findings underscore the importance of aligning therapeutic interventions with biologically relevant patient subsets rather than relying solely on syndromic definitions.

These observations ultimately informed the design of the TIGRIS trial, which prospectively tested the hypothesis that the therapeutic efficacy of PMX-HP depends on biologically defined patient selection^7)^. Unlike prior studies, TIGRIS deliberately restricted enrollment to patients with intermediate endotoxin activity (EAA 0.60-0.89) and high organ dysfunction, thereby operationalizing both predictive and prognostic enrichment. This approach reflects a broader paradigm shift in sepsis research toward precision medicine, in which treatment effects are expected to vary by underlying endotypes and host- response profiles^8, 9)^.

The recent full publication of TIGRIS as a multicenter, randomized, Bayesian phase 3 trial provides the rigorous evaluation of this strategy to date. Within this enriched population, polymyxin B hemoadsorption was associated with a high probability of improved survival, with posterior probabilities exceeding 95% at 28 days and 99% at 90 days^7)^. Notably, the treatment effect appeared more robust at 90 days than at 28 days, suggesting that the intervention may exert its primary benefit by modulating downstream host-response pathways rather than by immediate hemodynamic stabilization. This observation is consistent with current understanding of sepsis biology, in which persistent immune dysregulation, endothelial injury, and microvascular dysfunction contribute to late mortality^9, 10)^.

This temporal pattern is biologically plausible. Early mortality in septic shock is often driven by refractory circulatory failure, whereas later mortality is increasingly determined by unresolved organ dysfunction and secondary complications. By attenuating endotoxin-mediated signaling, PMX-HP may shift patients away from maladaptive inflammatory and thromboinflammatory trajectories, thereby improving longer-term outcomes. However, this interpretation remains inferential and requires further mechanistic validation.

At the same time, the interpretation of TIGRIS warrants careful consideration. The observed benefit at 28 days was sensitive to model specification and the incorporation of prior information, whereas the 90-day outcome was more robust across analytical approaches. This distinction highlights both the strengths and limitations of Bayesian trial design. While Bayesian frameworks allow the incorporation of prior evidence and improved efficiency in narrowly defined populations, they also introduce dependence on prior assumptions that must be evaluated transparently^11)^.

Importantly, the TIGRIS results do not resolve the longstanding challenges associated with endotoxin-targeted therapy; rather, they redefine the conditions under which such therapy may be effective. The data suggest that PMX-HP is unlikely to benefit unselected patients with septic shock but may have clinically meaningful effects in a specific endotype characterized by a moderate endotoxin burden and severe organ dysfunction. This concept is consistent with emerging evidence that treatment responsiveness in sepsis is governed by biological heterogeneity rather than clinical phenotype alone^8, 12)^.

Nevertheless, significant uncertainties remain. The endotoxin activity assay, although clinically available, is an indirect functional measure influenced by host immune status and assay characteristics, including potential saturation at high endotoxin levels^13)^. Furthermore, the feasibility of implementing EAA- guided strategies across diverse healthcare systems remains uncertain, particularly given issues related to assay availability, inter-center variability, and cost-effectiveness.

These considerations underscore the need to move beyond reliance on a single biomarker toward integrated approaches that incorporate pathogen burden, host-response profiling, and clinical severity. In this context, EAA should be viewed as one component of a broader stratification framework rather than a standalone decision tool. Future studies incorporating multimodal biomarkers, adaptive platform designs, and global validation cohorts will be essential to determine whether the signals observed in TIGRIS can be translated into consistent clinical benefit.

**Figure 2:**
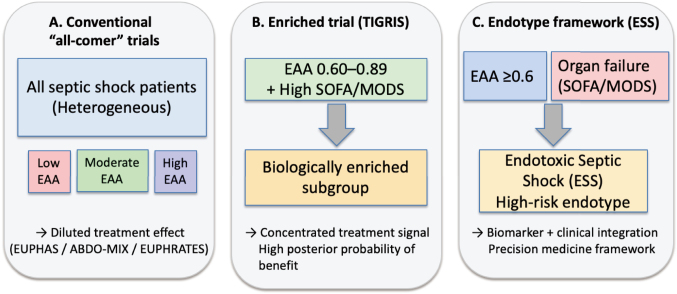
Why polymyxin B hemoperfusion trials diverged: the role of biological enrichment Panel A illustrates conventional “all-comer” trial designs, where heterogeneous septic shock populations dilute treatment effects. Panel B shows the TIGRIS approach, in which enrichment based on endotoxin activity assay (EAA 0.60-0.89) and high organ dysfunction concentrates treatment-responsive patients, resulting in a detectable survival signal. Panel C depicts the concept of endotoxic septic shock (ESS), defined by the combination of endotoxin activity and organ failure, representing a high-risk biological endotype. Together, these panels highlight how biomarker-guided enrichment can reveal therapeutic effects that remain obscured in unselected populations.

In this context, the TIGRIS trial represents an important step forward, not as definitive proof of efficacy, but as a demonstration that biologically informed trial design can reveal clinically meaningful signals that remain obscured in heterogeneous populations. (Table 1, Figure 2)

**Table 1:** Comparison of major RCTs of polymyxin B hemoperfusion in sepsis

Trial	Year	Design / Setting	N	Inclusion criteria	Primary endpoint	Main results	Key limitations
EUPHAS [1]	2009	Single-country (Italy), open-label RCT	64	Abdominal septic shock (post-surgery)	Hemodynamics + mortality (secondary)	Improved MAP, reduced vasopressor use; lower 28-day mortality	Small sample, early termination, no endotoxin measurement
ABDO-MIX [2]	2015	Multicenter (France), open-label RCT	243	Peritonitis-induced septic shock	28-day mortality	No mortality benefit	Incomplete treatments, no biological enrichment
EUPHRATES [3]	2018	Multicenter (North America), double-blind RCT	449	Septic shock with EAA ≥ 0.60	28-day mortality	No overall benefit; subgroup signal (EAA 0.60-0.89 with high MODS)	Heterogeneity, inclusion of very high EAA, delayed treatment
TIGRIS [5]	2025	Multicenter, Bayesian-adaptive RCT	157	Septic shock with EAA 0.60-0.89 + high severity	28- and 90-day mortality (Bayesian)	Posterior probability of benefit: 95.3% (28d), 99.4% (90d)	Preliminary data, small sample size

N, number of cases; RCT, randomized controlled trial; MAP, mean arterial pressure; EAA, endotoxin activity assay; MODS, multiple organ dysfunction syndrome

## Strengths of the EAA-guided approach

Several strengths of the EAA-guided approach deserve emphasis. First, it is grounded in a biologically coherent therapeutic rationale. Endotoxin is not merely a marker of infection burden but a central mediator of host-response dysregulation, driving cytokine amplification, endothelial activation, complement engagement, and immunothrombosis. These processes contribute directly to microvascular dysfunction and organ failure, providing a mechanistic basis for extracorporeal endotoxin removal^7, 10, 13)^.

Second, EAA-guided selection directly addresses the fundamental limitation of prior sepsis trials, biological heterogeneity. Increasing evidence indicates that septic shock comprises distinct endotypes with differential immune responses and treatment responsiveness, which cannot be adequately captured by syndromic definitions alone^8, 12, 14)^. The incorporation of EAA enables identification of a subgroup characterized by measurable endotoxin burden and high organ dysfunction, thereby aligning therapeutic targeting with disease biology^5, 7, 9, 15)^.

Third, the TIGRIS trial provides prospective validation of this enrichment strategy. By restricting enrollment to patients with EAA 0.60-0.89 and severe organ dysfunction, TIGRIS demonstrated a high posterior probability of mortality benefit, particularly at 90 days, supporting the concept that biologically informed selection can reveal treatment effects obscured in unselected populations^7, 9)^. This observation reinforces the interpretation that earlier neutral trials may reflect dilution of effect rather than absence of efficacy^6, 15, 16)^.

Fourth, the EAA offers practical advantages as a clinically deployable biomarker. Unlike transcriptomic or proteomic platforms, it can be performed rapidly at the bedside, facilitating time-sensitive decision-making in critically ill patients^8)^. Although its limitations are well recognized, its operational feasibility distinguishes it from many experimental precision-medicine tools.

Finally, the EAA-guided framework aligns with broader methodological advances in critical care research, including adaptive trial designs, Bayesian inference, and biomarker-driven enrichment strategies. These approaches have been increasingly advocated as necessary to improve signal detection and reduce failure rates in complex syndromes such as sepsis^8, 11, 14)^. In this regard, EAA-guided PMX- HP represents not only a therapeutic strategy but also a paradigm for pragmatically implementing precision medicine in the ICU^17)^.

## The limitations of EAA-guided patient selection

Despite its clinical utility, the EAA remains an imperfect biomarker, and its limitations must be carefully considered when used to guide therapeutic decisions. The EAA is an indirect functional assay that quantifies neutrophil oxidative burst in response to endotoxin-antibody complexes rather than directly measuring circulating endotoxin concentration^13, 18)^. Consequently, its performance is influenced not only by endotoxin burden but also by host immune competence, including neutrophil function, immune suppression, and prior immunomodulatory therapy. In critically ill patients, particularly those with sepsis-induced immunoparalysis or neutropenia, EAA values may underestimate biologically relevant endotoxemia, thereby introducing the risk of misclassification^19, 20)^.

A further limitation relates to the non-specificity of endotoxin activity. Elevated EAA levels are not restricted to Gram-negative infections and may also occur in Gram-positive sepsis, fungal infections, or sterile inflammatory conditions, likely reflecting gut translocation or systemic inflammatory amplification^21-23)^. This lack of pathogen specificity complicates interpretation and raises the possibility that EAA reflects a broader host-response phenotype rather than a purely pathogen-driven signal.

In addition, the dynamic nature of endotoxin release poses important challenges. Endotoxemia is not static but fluctuates over time in response to infection control, antimicrobial therapy, and host-pathogen interactions. A single time-point measurement may therefore fail to capture clinically relevant trajectories, and serial assessment may be required to improve diagnostic accuracy and patient selection^24, 25)^. This issue is particularly relevant in early sepsis, where endotoxin levels may evolve rapidly and therapeutic windows are narrow.

Technical constraints of the assay must also be acknowledged. At high endotoxin levels (EAA ≥ 0.90), the assay approaches saturation, limiting its ability to discriminate between extreme values^13)^. This phenomenon likely contributed to the absence of treatment effect observed in patients with very high EAA in the EUPHRATES trial, suggesting that such patients may represent a biologically distinct subgroup with either overwhelming endotoxemia beyond the capacity of adsorption therapy or fundamentally different pathophysiology^6, 26)^.

From a translational perspective, reliance on EAA as a standalone gatekeeper risks oversimplification of a complex biological process. Sepsis pathophysiology involves the interplay of pathogen burden, host immune response, endothelial dysfunction, coagulation activation, and metabolic reprogramming. A single biomarker is unlikely to fully capture this multidimensional landscape. Emerging evidence supports integrating endotoxin measurements with complementary biomarkers, such as cytokines, endothelial injury markers, and NETosis-related parameters, to improve phenotypic resolution and therapeutic targeting^10, 27)^.

Finally, practical considerations must be addressed. The availability of EAA testing varies across institutions, and inter-laboratory variability, turnaround time, and cost may limit widespread implementation. These factors are particularly relevant in resource-limited settings and may affect the generalizability of EAA-guided strategies.

Taken together, these limitations highlight that while EAA is a valuable tool for enrichment, it should be interpreted within a broader clinical and biological context. Future strategies should focus on integrated, multimodal phenotyping approaches that combine pathogen-specific signals, host-response biomarkers, and clinical severity indices to achieve more reliable and reproducible patient stratification.

## Toward integrated strategies

The limitations of single-biomarker approaches underscore the need for more comprehensive strategies to guide patient selection in septic shock. Increasing evidence suggests that sepsis is not a uniform syndrome but a constellation of biologically distinct endotypes defined by the interaction between pathogen burden and host response. In this context, reliance on endotoxin activity alone is unlikely to fully capture the complexity of disease biology or predict therapeutic responsiveness. Instead, a multimodal approach integrating pathogen-derived signals, host-response biomarkers, and clinical phenotyping is required to achieve meaningful precision in patient stratification.

Combining EAA with established biomarkers of inflammation and infection, such as interleukin-6, procalcitonin, and presepsin, may enhance discrimination between hyperinflammatory and immunosuppressed states. At the same time, markers of endothelial injury (e.g., angiopoietin-2, soluble thrombomodulin) and coagulation activation may provide complementary information regarding microvascular dysfunction, a key determinant of organ failure in sepsis^10, 28)^. Such integrated biomarker panels have the potential to better define biologically coherent subgroups than any single parameter alone.

Advances in transcriptomic profiling further support this paradigm. Several studies have identified reproducible sepsis endotypes characterized by distinct immune signatures and differential responses to therapies, including corticosteroids^12, 29)^. These findings suggest that treatment effects are contingent on underlying molecular phenotypes, reinforcing the need for biologically informed enrichment strategies in clinical trials. More recently, machine learning approaches integrating clinical variables with multi-omics data have demonstrated improved predictive performance for outcomes and treatment response, highlighting the potential of data-driven phenotyping in critical care^30)^.

In parallel, adaptive and platform trial designs provide a methodological framework for efficiently testing such stratified approaches. By allowing dynamic modification of enrollment criteria and treatment allocation based on interim biological signals, these designs can better accommodate the heterogeneity inherent in sepsis and accelerate the identification of responsive subgroups^31)^. The Bayesian framework used in TIGRIS represents one step in this direction, but further integration with biomarker-driven adaptive strategies will likely be required to fully realize the potential of precision medicine in sepsis.

Importantly, translating integrated strategies into clinical practice must consider feasibility and scalability. While multi-omics platforms provide deep biological insight, their complexity and cost may limit immediate bedside applicability. Therefore, a pragmatic approach may involve tiered stratification, in which readily available biomarkers such as EAA and routine laboratory parameters are used for initial screening, followed by more detailed molecular characterization in selected populations. This stepwise model may balance precision with practicality and facilitate broader implementation.

Ultimately, the goal is to move from reductionist, single-marker paradigms toward systems-level phenotyping, where multiple dimensions of sepsis biology are incorporated into decision-making^32)^. In this framework, EAA remains valuable, not as a definitive selector, but as one component within a broader, integrated strategy. Future studies should focus on validating such multimodal approaches across diverse populations and healthcare settings, with particular attention to reproducibility, cost- effectiveness, and clinical impact.

## Global perspectives and implementation challenges

Clinical adoption of PMX-HP has been markedly heterogeneous across regions, reflecting differences in clinical practice patterns, regulatory environments, and interpretation of the evidence base. In Japan, PMX-HP has been incorporated into routine clinical care and is supported by national reimbursement systems, with registry and observational data suggesting improvements in hemodynamics and, in selected populations, survival^33)^. In contrast, uptake in Europe and North America has remained limited, largely due to neutral findings from earlier randomized trials and the absence of consistent confirmatory evidence in broader septic shock populations^2, 3)^.

These geographic discrepancies highlight a central issue: the tension between real-world practice and randomized evidence. Observational studies often include patients treated earlier in the disease course or selected based on clinician judgment, whereas randomized trials have historically enrolled heterogeneous populations without biological enrichment. The emergence of TIGRIS and similar enrichment- based approaches may help reconcile these differences by identifying subgroups more likely to benefit, but external validation across diverse healthcare systems remains essential^7, 34)^.

From an implementation perspective, several practical challenges must be addressed. PMX-HP requires specialized equipment, trained personnel, and coordination with extracorporeal circulation platforms, which may limit accessibility in resource- constrained settings. In addition, the requirement for EAA testing introduces further complexity, including assay availability, turnaround time, and inter-laboratory variability^13)^. These factors may affect both feasibility and reproducibility, particularly outside high-resource ICUs.

Economic considerations are equally critical. Extracorporeal therapies are inherently resource- intensive, and cost-effectiveness analyses have suggested that PMX-HP is most likely to be economically justified only in narrowly defined high-risk populations. This reinforces the importance of precise patient selection and supports the rationale for biomarker-guided strategies to maximize therapeutic efficiency^35, 36)^. Without such targeting, widespread implementation may not be sustainable within most healthcare systems.

Another important consideration is the timing of intervention, which may vary across healthcare systems. Delays in diagnosis, differences in ICU admission thresholds, and variability in sepsis management pathways can all influence the window during which endotoxin removal may be effective. Early identification of appropriate candidates, potentially in the emergency department or early ICU phase, may therefore be critical for optimizing outcomes^37)^.

Taken together, these considerations underscore that the future of PMX-HP will depend not only on biological plausibility and trial results but also on feasibility, scalability, and health-system integration. Bridging the gap between controlled trial settings and real-world clinical practice will require pragmatic trials, implementation research, and region-specific strategies that account for differences in healthcare infrastructure and resource availability.

## Looking ahead: The road to regulatory and clinical acceptance

The TIGRIS trial and related analyses represent an important step forward in evaluating PMX-HP, but they do not yet constitute definitive evidence sufficient for broad regulatory approval or universal clinical adoption. A key next step will be external validation in larger, geographically diverse populations, as the highly selected cohort enrolled in TIGRIS may not reflect the full spectrum of septic shock encountered in routine practice. Replication across different healthcare systems, including varying case-mix, treatment timing, and resource availability, will be essential to establish generalizability and robustness of the observed treatment effect^34, 38)^.

Future clinical trials should build on the enrichment strategy employed in TIGRIS while addressing its limitations. In particular, adaptive platform trials offer a promising framework to evaluate PMX-HP within a broader ecosystem of sepsis therapies. Such designs allow simultaneous testing of multiple interventions across biologically defined subgroups, with dynamic modification of enrollment criteria based on accumulating evidence^31, 39)^. This approach may be particularly well suited to sepsis, where heterogeneity and time-dependent treatment effects have historically limited the success of conventional randomized controlled trials.

In parallel, regulatory acceptance will increasingly depend on integrating biomarker-driven evidence with clinically meaningful endpoints. While 28-day mortality has traditionally served as the primary endpoint in sepsis trials, there is growing recognition that longer-term outcomes, including 90-day survival, organ dysfunction trajectories, and patient- centered measures such as quality of life, may better capture the true impact of interventions^32, 40)^. The consistent signal observed at 90 days in TIGRIS aligns with this evolving perspective and may support a shift toward more biologically and clinically relevant endpoints in future studies.

Another critical issue is the standardization of patient selection criteria. Although EAA provides a practical tool for identifying endotoxin-associated endotypes, variability in assay performance, cutoff definitions, and measurement timing may affect reproducibility across studies. Harmonization of biomarker thresholds and incorporation of complementary markers will be necessary to ensure consistency and facilitate regulatory evaluation^13, 41)^.

Health technology assessment will also play a central role in determining the future of PMX-HP. Demonstrating cost-effectiveness in well-defined patient populations will be essential for reimbursement decisions, particularly in healthcare systems with constrained resources. This will require prospective studies that incorporate economic endpoints alongside clinical outcomes, as well as real-world data, to assess implementation feasibility and scalability^35, 42)^.

Finally, the broader success of PMX-HP will depend on its integration into a comprehensive precision medicine framework for sepsis. Rather than being evaluated in isolation, endotoxin removal should be considered alongside other targeted therapies addressing inflammation, coagulation, endothelial dysfunction, and immune dysregulation. Advances in systems biology, machine learning, and multimodal biomarker integration provide an opportunity to develop individualized treatment strategies that align therapeutic interventions with patient-specific pathophysiology^29, 43)^.

In this context, the path forward for PMX-HP is likely to involve iterative refinement rather than binary validation. Continued convergence of biological insights, innovative trial design, and pragmatic implementation strategies will be required to determine whether the promising signals observed in enriched populations can be translated into durable, generalizable clinical benefit.

## Conclusion

This article offers a timely and thoughtful perspective on the evolving role of EAA-guided PMX-HP in the treatment of septic shock. It underscores the importance of precision medicine in critical care and demonstrates how revisiting prior "failed" therapies through biomarker enrichment can yield new insights. Yet, it also reminds us of the perils of over-reliance on a single imperfect biomarker. The path forward lies in integrating assays, clinical phenotypes, and molecular signatures within adaptive, precision-guided frameworks. Whether PMX- HP ultimately secures a place in the therapeutic arsenal for sepsis will depend on our ability to translate these principles into reproducible, globally applicable, and economically sustainable practice.

## Author contributions

RF and TI wrote the draft, and KN, SU, HM, and NU significantly reviewed the manuscript. All authors read and approved the final manuscript.

## Conflicts of interest statement

The authors declare that they have no conflict of interest. TI is a member of the JMJ editorial board and were not involved in the peer review or decision-making process for this paper.
